# Multi-plane remote refocusing epifluorescence microscopy to image dynamic ${\mathrm{Ca}}^{2+}$ events

**DOI:** 10.1364/BOE.10.005611

**Published:** 2019-10-10

**Authors:** Penelope F. Lawton, Charlotte Buckley, Chris D. Saunter, Calum Wilson, Alexander D. Corbett, Patrick S. Salter, John G. McCarron, John M. Girkin

**Affiliations:** 1 Department of Physics, Durham University, South Road, Durham, DH1 3LE, UK; 2 Strathclyde Institute of Pharmacy & Biomedical Sciences, University of Strathclyde, 161 Cathedral Street, Glasgow, G4 0RE, UK; 3 Department of Physics, University of Exeter, North Park Road, Exeter, EX4 4QL, UK; 4 Department of Engineering Science, University of Oxford, Parks Road, Oxford OX1 3PJ, UK

## Abstract

Rapid imaging of multiple focal planes without sample movement may be achieved through remote refocusing, where imaging is carried out in a plane conjugate to the sample plane. The technique is ideally suited to studying the endothelial and smooth muscle cell layers of blood vessels. These are intrinsically linked through rapid communication and must be separately imaged at a sufficiently high frame rate in order to understand this biologically crucial interaction. We have designed and implemented an epifluoresence-based remote refocussing imaging system that can image each layer at up to 20fps using different dyes and excitation light for each layer, without the requirement for optically sectioning microscopy. A novel triggering system is used to activate the appropriate laser and image acquisition at each plane of interest. Using this method, we are able to achieve axial plane separations down to 15 μm, with a mean lateral stability of ≤ 0.32 μm displacement using a 60x, 1.4NA imaging objective and a 60x, 0.7NA reimaging objective. The system allows us to image and quantify endothelial cell activity and smooth muscle cell activity at a high framerate with excellent lateral and good axial resolution without requiring complex beam scanning confocal microscopes, delivering a cost effective solution for imaging two planes rapidly. We have successfully imaged and analysed ${\mathrm{Ca}}^{2+}$ activity of the endothelial cell layer independently of the smooth muscle layer for several minutes.

## 1. Introduction

The ability to image at increasingly higher frame rates to capture rapid biological processes is a continuing goal for scientists across disciplines. Frequently this is achieved via imaging in epifluorescence in a single focal plane, using a single excitation wavelength. By eliminating any moving parts, images can be acquired at up to the maximum frame rate of the camera and the limit of brightness of the image. However, many studies require the acquisition of more than one focal plane to understand biological processes and communication, and increasingly using a range of wavelengths to excite different fluorophores.

Changing the focal depth of a microscope system may be accomplished in several ways. Most easily, the sample itself may be moved up and down. However, this creates a disturbance of the sample, may shift the sample laterally, and is generally slow with respect to many rapid biological events. Another method for adjusting the focal depth is via movement of the microscope objective. Microscope objectives are, however, large and bulky, particularly at high numerical apertures, and while methods of moving them have been improved [1] vibrations from moving a heavy microscope objective may also disturb the sample. This is also impractical in the case of oil and water immersion objectives, as the immersion media may distort the sample coverslip during the movement of the objective lens, potentially leading to image aberrations and sample movement [2].

Methods which carry out the refocussing in another area of the optical setup are generally faster and cause less sample disturbance. One such method is via the use of adaptive optical elements, such as deformable mirrors [3], tunable lenses [4] and spatial light modulators [5]; these have been used to correct defocus aberrations at depth in biological specimens and may also be used for active focus locking in confocal microscopy [6]. Adaptive optical elements, however, add another layer of complexity to the optical train and may be accompanied by a loss of light due to the diffraction efficiency of these elements.

An elegant way to avoid this is to re-image the specimen through the use of a second microscope objective [7]. This approach was originally designed to eliminate sample disturbance for techniques such as fast confocal microscopy [8]. Refocussing re-images the focal region of the first microscope objective at a second microscope objective without moving either objective, creating an image with minimal aberrations. This second image then needs to be magnified onto the camera, which can be done with either an identical third objective or through the same second objective through the use of a retro-reflecting mirror. Here, a mirror is coupled to a motor to axially translate the imaging focal volume. With a fast enough motor the system may be run at high speed limited only by the integration time of the camera required for a suitable image.

One biological system in which the ability to acquire rapid multiplane images is advantageous is in blood vessels. Arteries comprise several main cell layers; those of particular interest are the inner endothelial cell layer which is in contact with, and processes signals from, the blood. This inner layer then transmits signals to the outer smooth muscle layer through the propagation of ${\mathrm{Ca}}^{2+}$. The signalling process is vital for a wide range of physiological functions, informing the smooth muscle cells when to contract and relax [9]. However, how this signal transduction occurs remains poorly understood, in part due to the difficulties in imaging signalling events between the two layers rapidly enough. When imaging ${\mathrm{Ca}}^{2+}$ signals, a low excitation light level is needed to limit altering biological processes. Significantly, even in a widefield epi-illumination microscope, a high magnification, high NA objective lens provides a small enough axial point spread function (PSF) to resolve endothelial and smooth muscle layers independently of each other, rendering confocal or multiphoton light excessive and unnecessary for this application, respectively.

In this paper we characterise an epifluorescence-based remote refocussing microscope setup and validate it using intact *en face* arterial preparations to image endothelial ${\mathrm{Ca}}^{2+}$ signalling. The system is configured to re-image the focal volume acquired with a 60X, 1.4NA oil objective into a second 60X, 0.7NA long working distance air lens and onto a retro-reflecting mirror coupled to a motor. To define specific focal planes, optical switches connected to the motor are used to output TTL voltages to trigger the camera and the individual laser lines at user-defined z-planes. Using this microscope setup we were able to cleanly resolve smooth muscle and endothelial cell layers separately at up to 20fps in live rat carotid preparations and image ${\mathrm{Ca}}^{2+}$ transients in the endothelial layer distinct from the smooth muscle layer.

## 2. Methods

### 2.1. Optical setup

**Fig. 1.**
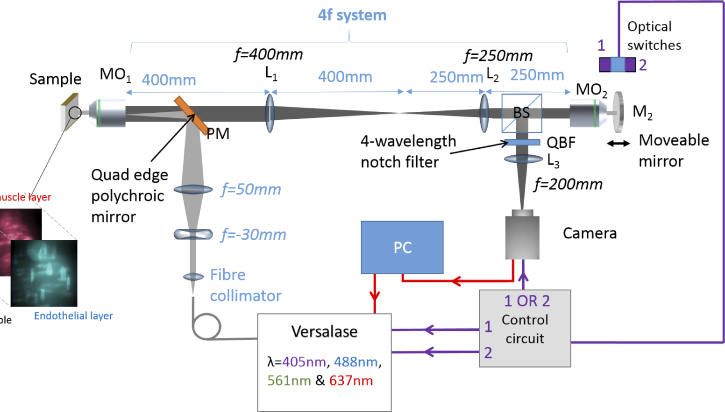
A schematic of the experimental setup. ${\mathrm{MO}}_{1}$ = 60x 1.4NA Plan Apo oil immersion microscope objective (Nikon), $L_{1}$ = 400mm lens, $L_{2}$ = 250mm lens, BS = 50:50 beamsplitter, ${\mathrm{MO}}_{2}$ = 60x 0.7NA air Plan Fluor microscope objective with a correction collar (Nikon), $M_{2}$ = moving mirror, PM = polychroic mirror reflective at 405,488,561 and 635 nm (Laser 2000), QBF = quad band 405-488-561-640 nm emission filter (Chroma Inc.)

The samples were imaged in widefield epifluorescence mode (Fig. 1) using a four wavelength Stradus Versalase system (Vortran Laser Technology) for illumination. This allows for spectrally distinct fluorophores specific to different tissue layers to be illuminated by their respective laser wavelengths, increasing the contrast between images of endothelial and smooth muscle cell layers. The laser is triggered via two optical switches connected to two separate laser modules within the Versalase system. The optical switches are operated via a slotted plate coupled to the mirror $M_{2}$. Activation of the optical switch at specific points activates the laser output only when the system is focussed on a certain sample plane.

The fiber output from the laser system is collimated, then focussed into the back of a microscope objective ${\mathrm{MO}}_{1}$ (Nikon 60X 1.4NA oil objective). This gives a collimated beam at the sample, and thus all planes of the sample are similarly illuminated using widefield illumination. Fluorescence light passes back through a polychroic mirror with high reflectivity at 405,488,561 and 635 nm (Laser 2000) and a quad band emission filter with low transmission at 405,488,561 and 640 nm (Chroma Inc.). The light is subsequently reimaged onto the back of the second microscope objective ${\mathrm{MO}}_{2}$ (Nikon 60X 0.7NA air objective) via a 4f lens system. The lenses used have focal lengths of 400mm and 250mm, providing a pupil demagnification of 1.6. This results in a slight mismatch of the pupil sizes between the air and oil objectives; according to [7], the overall magnification from the sample plane to the remote objective plane must equal the ratio of the refractive indices. For the objectives used here, with the same magnification and focal length, the demagnification of the primary pupil should be 1.51, the refractive index of oil. A pupil mismatch would not normally be used in remote refocussing as this can lead to aberrations, reducing the lateral and axial resolution; however, to image the full depth of endothelial cells, a lower axial resolution is an advantage. The optical plane at the sample is thus recreated at the focus of ${\mathrm{MO}}_{2}$, and the required plane may be selected through the movement of the mirror $M_{2}$ rather than through movement of the sample. The light plane reflected by the mirror then passes back through a beamsplitter and tube lens into the Photometrics Cascade 512b camera. The camera shutter is triggered using the optical switches to acquire images depending on the position of the mirror $M_{2}$, using the slotted optical switch signal from the moving mirror. Images are acquired using Micromanager 1.4.22. [10].

### 2.2. Motor and cam mechanism

**Fig. 2.**
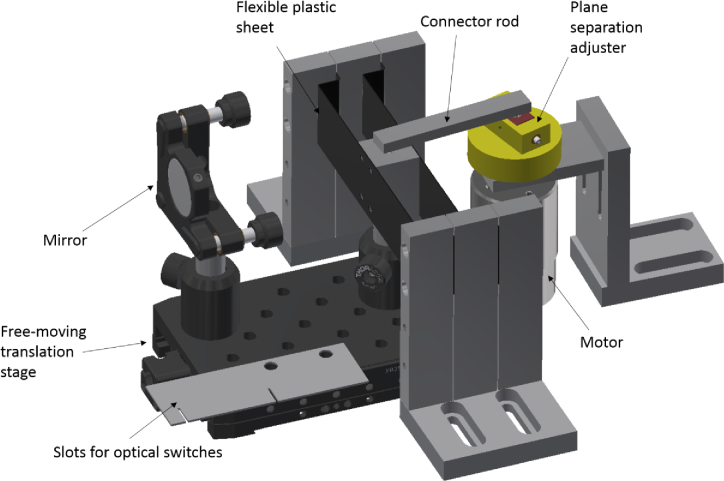
A schematic of the mechanism used to move the refocusing mirror rendered in Autodesk Inventor. The plane adjustment mechanism is highlighted in yellow and red.

The refocusing mirror is moved via a cam mechanism, as shown in Fig. 2. The mirror is mounted onto a translation stage which has been modified to move freely. This mirror is coupled to an aluminium block attached to two plastic flexures. These flexures, along with the stage, restrict the motion of the mirror to one dimension parallel to the optical axis. The aluminium block is coupled to a rigid aluminium rod, which is coupled to the motor (Globe Motors brushed DC motor, max RPM 5200, manufacturer number 403A6005-2) via the plane adjustment mechanism (highlighted in yellow and red in Fig. 2). The red block may be moved backwards and forwards using finely threaded screws on either side, moving the centre of rotation of the aluminium rod away from the axis of rotation of the motor and thus acting as an adjustable eccentric cam, with the rod as the follower. Thus, the motion of the mirror, and the distance between the focal planes, can be accurately controlled by changing the position of the red block. A thin metal plate with two slots is also attached to the stage. The slots are placed between the optical switches precisely to either block or reveal either switch when the mirror is at either extreme of its motion. The output of each optical switch is then connected to the inputs of an OR gate (Toshiba TC74HC32AP(F)), and the output was connected to the camera. The separate outputs of the optical switches were also connected to separate laser module inputs in the Stradus Versalase system.

### 2.3. System triggering

**Fig. 3.**
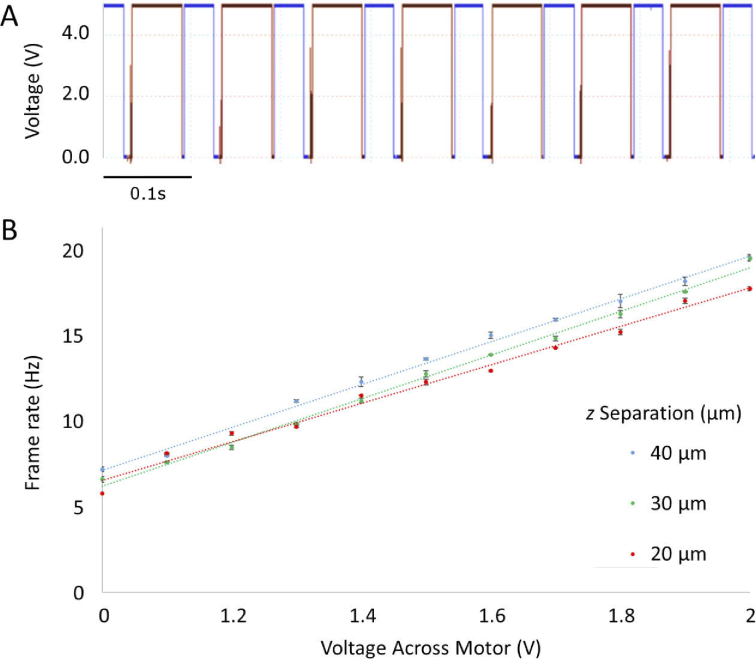
(A) Representative TTL trace from optical switches on the oscilloscope. The camera and corresponding laser triggers off the rising edge of each pulse. The red and blue traces indicate different optical switch outputs (B) Relationship between imaging frame rate (Hz) and voltage across the motor (V) at different z separations. Increasing voltages lead to a linear increase in acquisition frame rate. Increasing the voltage any higher than approximately 2V resulted in the camera being unable to acquire the images rapidly enough.

The triggering of the camera and the laser are controlled by TTL pulses produced by the two slotted optical switches (Honeywell HOA-2001 transmissive Optoschmitt sensor). The switches comprise an infrared LED and a photodiode which outputs to a transistor and 10 kΩ pull up resistor, outputting a TTL signal when the switch is unblocked and zero signal out when the switch is blocked. A slotted metal plate is used to block and unblock the switches. The plate is coupled to the mirror $M_{2}$, which in turn is coupled to a motor. As the motor turns, the stage moves backwards and forwards via a simple cam mechanism, moving the slotted plate to unblock the relevant switches when the mirror is at each extreme of its motion. Thus the output of the circuit is only ‘ON’ when the system is focussed at a single plane. A representative oscilloscope trace from the optical switches is shown in Fig. 3(A). The speed of the motor itself, and hence the speed of the refocussing system, is controlled by altering the voltage across it, leading to a linear increase in frame rate (Fig. 3(B)). The output TTL signals from the optical switches can be connected to a specific laser line output on the Versalase control box, selected depending on which fluorophores are being imaged at each axial plane. The pulses are also connected to the inputs of an electronic OR gate, which outputs a TTL pulse whenever either of the switches outputs a TTL pulse. This output is connected to the rear of the camera and thus acquisition only occurs at the planes required for imaging, coinciding with the laser activation.

### 2.4. Calibration with a fluorescent etched sample

**Fig. 4.**
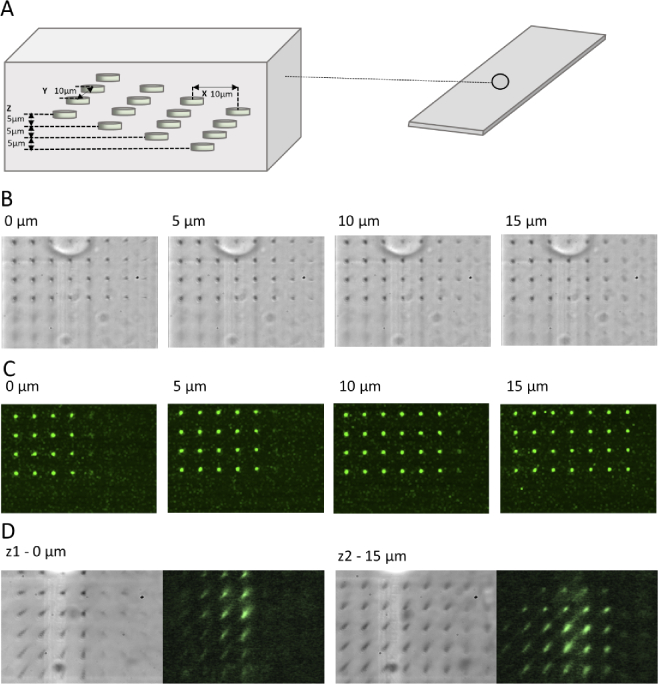
A femtosecond laser-etched [11,12] calibration sample was used to calibrate lateral pixel size and axial plane separation. (A) A diagram of the volume used for x-y-z calibration. The femtosecond-etched features are spaced in a 10 μm regularly-spaced grid and start at successive depths of 5 μm into the sample. (B) Example images were taken every 5 μm over a 15 μm axial range in transmission and fluorescence for the features to demonstrate this. (C) Confocal images of the slides taken using a separate apparatus using 488 nm light (D) Images taken with our setup using 488 nm light, compared here to their transmission appearance 15 μm apart.

The pixel size and axial image separation were determined using a specially designed femtosecond laser-etched sample slide as shown in Fig. 4 [12,11]. The calibration specimen was composed of a 1 mm thick sheet of acrylic into which was embedded a well defined array of features using a direct laser writing process detailed in [13]. In summary, the output beam from an amplified Ti:Sapphire laser (790 nm, 250 fs, 1 kHz rep rate, Solstice, Spectra-Physics) was intensity modulated by a half wave plate in a motorised mount. To compensate for aberrations introduced by the index mismatch between the immersion oil of the fabrication objective and acrylic substrate, the beam was then expanded and relayed onto a spatial light modulator (SLM, Hamamatsu X10468-02). The SLM was in turn imaged in a 4f configuration onto the back focal plane of the fabrication objective lens (Olympus 60X 1.4 NA). Phase corrections (predominantly spherical) provided by the SLM were made using a sensorless adaptive optics approach in a Zernike basis, with a metric related to the threshold for laser fabrication [14]. The patterns themselves were composed of a three-dimensional array of features on a 10 μm square grid at each depth and a 5 μm axial separation. The lateral extent of the grid of features changed with each depth such that the precise depth could be determined uniquely from the number and arrangement of the features. 10 individual layers were written, with the number limited only by the working distance of the fabrication lens.

This slide was then used to calibrate our system and measure the distance between planes. To increase or decrease the separation of the planes being imaged, a micrometer screw was used to adjust the distance between the axis of rotation of the motor and the axis coupling the motor to the mirror. A reference axial separation dataset was acquired every 5 μm by manually moving the imaging microscope objective MO${\mathrm{MO}}_{1}$ in 5 μm steps (Fig. 4(B)). These images were compared to reference images taken using a standard confocal microscope of the sample volume (Fig. 4(C)) to check that the pattern of features was as expected. Using this approach, it was possible to image two planes using the optical switch-generated TTL pulses, and determine the relative axial separation. For example, the separation of the images shown in the corresponding brightfield and fluorescence images in Fig. 4(D) is 15 μm. All axial separation distances were determined in this way.

### 2.5. Stability measurements

The system was set up to image two different depth planes labelled $z_{1}$ and $z_{2}$ of the sample slide described in Fig. 4 in brightfield, with $z_{2}-z_{1}$=15 μm. The ability of the system to accurately re-image the same plane at subsequent time points was then tested over both long (5000 frames/plane) and short (50 frames/plane) timescales. The stability for fast (20fps) and slow (10fps) movement of the refocussing mirror was also measured. Images were processed in Python using the ‘trackpy’ Python package [15]. This involved locally thresholding the stacks, allowing the lateral position of the point-like sample features to be determined. The positions were recorded for each image in the stack and the average displacement of each feature position from the mean position was recorded, as well as the feature trajectories.

### 2.6. Mean apparent spot separation

To calculate the mean apparent spot separation (MASS), 100 images of the calibration slide dots were acquired in fluorescence mode at 5 μm intervals from z=0μm to z=100μm. An average intensity projection was performed in Fiji [16] on each stack and a line profile taken across the same pair of vertical and horizontal dots at each z position. These line profiles were plotted in Matlab and the distance between the peaks calculated, which were then plotted in Matlab along with the averages across the z range.

### 2.7. Arterial preparations

**Fig. 5.**
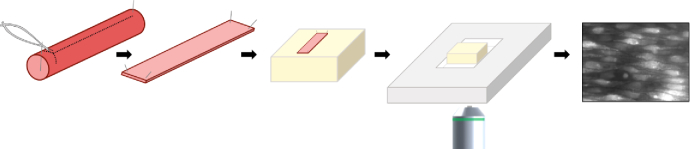
Rat carotid arteries were dissected, opened longitudinally using microscissors and the artery pinned out on a Sylguard block with the endothelium exposed. Arteries were then stained with Cal-520 (5 uM) with 0.02% pluronic F-127 for 30 mins at 37°C. After washing, preparations were placed face down on a 0 grade thickness coverslip fixed to the bottom of a custom bath chamber (3cm x 1.5cm) on 200 μm diameter stainless steel pins to stop the preparation touching the coverslip. Preparations were kept in PSS at all times.

All animal care and experimental procedures were carried out with the approval of the University of Strathclyde Local Ethical Review Panel [Schedule 1 procedure; Animals (Scientific Procedures) Act 1986, UK], under UK Home Office regulations. Male Sprague-Dawley rats (10-12 weeks old; 250-350g) were euthanized by an overdose of pentobarbital sodium (200 mg kg-1, I.P.) and the carotid arteries dissected out and kept in PSS (physiological saline solution: 145 mM NaCl, 2 mM MOPS, 4.7 mM KCl, 1.2 mM NaH2PO4, 5 mM Glucose, 0.02 mM EDTA, 1.17 mM MgCl, 2 mM CaCl, pH 7.4). Arteries were pinned out on Sylgard blocks, cut open along the length of the artery and pinned out flat with the endothelial cells exposed, as described in [17,18] and shown in Fig. 5. For imaging fixed arteries, preparations were incubated in 70 % EtOH for 10 mins, washed and incubated in 5 μM SytoGreen and 1.5 μM PI (Propidium Iodide) for 30 mins to stain both endothelial and smooth muscle cell nuclei, then washed thoroughly and mounted for imaging.

For live imaging of arteries, preparations were incubated in either Cal-520 (5 μM) calcium indicator dye alone, or both Cal-520 and TMRE (120 nM) mitochondrial indicator dye, with 0.02% Pluronic F-127 for 30 mins at 37°C. Preparations were washed thoroughly in PSS and mounted for imaging. To stimulate endothelial Ca^2+^ signalling, acetylcholine (ACh) (0.5 μM) was applied to preparations during imaging.

The Sylgard blocks with the prepared arteries were placed face down on 0 grade thickness microscope coverslips fixed to custom-made bath chambers with 200 μm diameter stainless steel pins used as spacers. This ensured endothelial cells did not come into contact with the coverslip, and allowed space for flow.

### 2.8. Imaging setup and parameters

Image acquisition was performed through Micromanager v1.4.22 [10], with camera gain set at 4.09. Brightfield illumination, provided by a Nikon white light source, was acquired with an exposure of 1ms, whilst epifluorescence illumination, provided by the Vortran Versalase at 1% power (to minimise photobleaching), was acquired with 10ms exposures. The optical switch TTL output was monitored through a Picoscope (Pico Technology), and the switches set up such that the trace resembled that in Fig. 3(A). The speed of the triggering was set by changing the voltage across the DC motor driving the mirror motion, which showed a linearly proportional relationship to the frame rate, as shown in Fig. 3(B). The camera shutter was set to External Triggering (Strobed) to acquire images at the two planes of interest. To trigger the Versalase system on the same TTL pulses, the Versalase was connected to the computer via an RS-232 connection and set to External Control in the Versalase software, and each laser line to one of the electronics board of an optical switch via a BNC cable. This flexibility allowed us to change the laser line associated with each switch, and the laser line with which each plane was imaged.

### 2.9. Image processing

Images were all processed in Fiji. To obtain ACh-induced Ca^2+^ traces from endothelial cells, a standard ROI was applied to each cell manually in Fiji and the signal intensity measured over time. A rolling average of signal intensity was calculated across 10 measurements to smooth signals, and the $F/F_{0}$ value calculated from a baseline average of the first 10 images. The traces for each cell were baseline corrected in Matlab to remove bleaching, and the signals equally spaced in *y* to visualize individual traces.

## 3. Results

### 3.1. System resolution

**Fig. 6.**
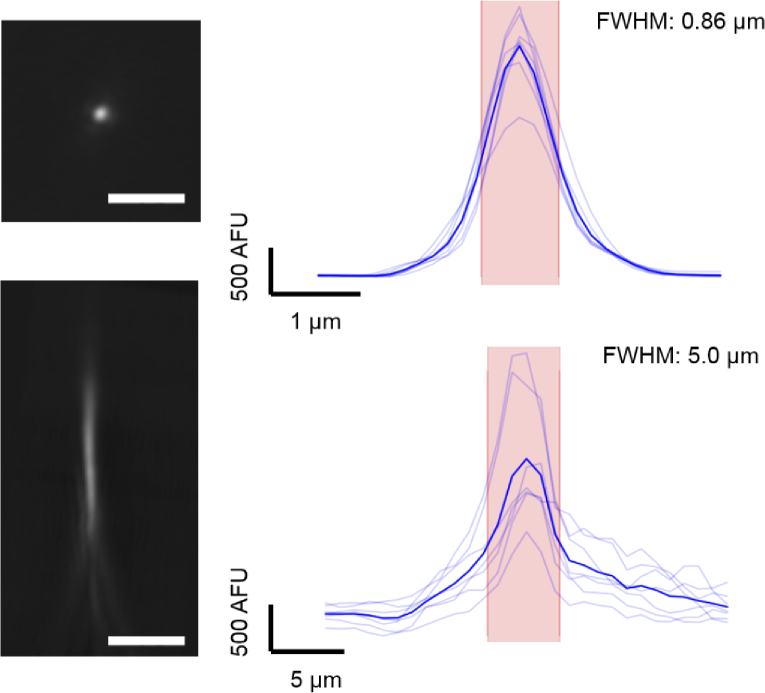
(a)0.5 μm diameter fluorescent beads were imaged as single planes for the lateral resolution (top panel), and at Δz = 1 μm intervals for the axial resolution (bottom panel). Images were deconvolved with a theoretical PSF, then a line profile was taken across their diameter using Fiji and plotted (light blue) for each bead analysed. The mean was plotted (dark blue) and the FWHM (width of the red area) calculated. Scale bar is 5 μm.

Imaging sub-diffraction limited beads (FocalCheck Test Slide, F36909) and then deconvolving these images with a predicted PSF in Fiji showed that the lateral and axial resolution of the system, as calculated using the FWHM, are 0.87 μm and 5.0 μm, (Fig. 6(a)). The lowest numerical aperture objective used in the system, the LWD 60X 0.7NA air objective lens, has a theoretical lateral resolution of 350 nm and axial resolution of 2 μm at 500 nm. As this objective images a 1.6x larger version of the sample due to system magnification, the effective theoretical resolution of the system is therefore 218 nm laterally and 1.25 nm axially; about 4 times smaller than that measured. The resolutions of each objective were also checked separately. The higher numerical aperture objective has a lateral and axial resolution of 180nm and 510nm respectively. Experimentally, the lateral and axial resolutions were found to be 239nm and 1.47 μm respectively, which are around 2-3 times larger the theoretical values. The lower numerical aperture objective was also checked outside the system, and lateral and axial resolutions of 486nm and 4.9 μm were measured experimentally, which are again 2-3 times larger than the theoretical values. The discrepancy in our system is therefore most likely due to system aberrations and the objectives themselves, but is ideally suited for our experimental requirements. These measured resolution values are well within the regime required to image Ca^2+^ transients in cells of approximately 100 μm length, 15 μm apart.

### 3.2. System characteristics

#### 3.2.1. Mean apparent separation with depth

**Fig. 7.**
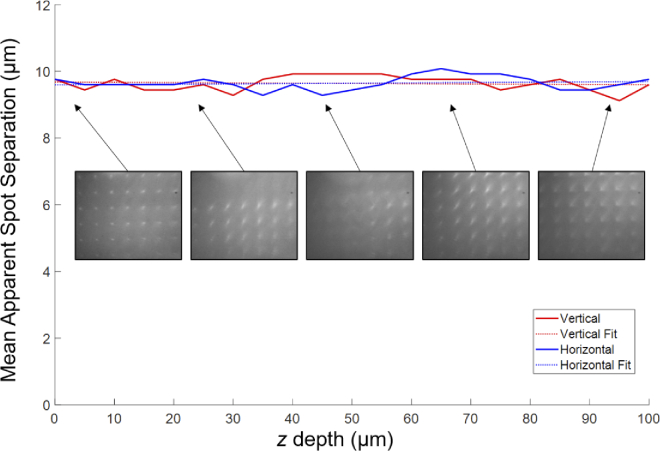
The calibration slide in fluorescence mode was imaged every 5 μm and the mean apparent spot separation (MASS) calculated for a vertical (red) and horizontal (blue) spot pair (solid line). The mean value was also plotted (dashed line), and example images of the calibration slide shown from 0, 20, 45, 70, 95 μm depths.

To ensure that the system maintained equal magnification with depth, the calibration slide was imaged in fluorescence mode every 5 μm through 100 μm depth. The vertical and horizontal mean apparent spot separation (MASS) were measured for a spot pair and plotted with the mean (Fig. 7). We found no systematic or significant difference in the MASS with depth, giving confidence that the system maintains equal magnification with depth.

#### 3.2.2. Reproducibility of axial plane position

**Fig. 8.**
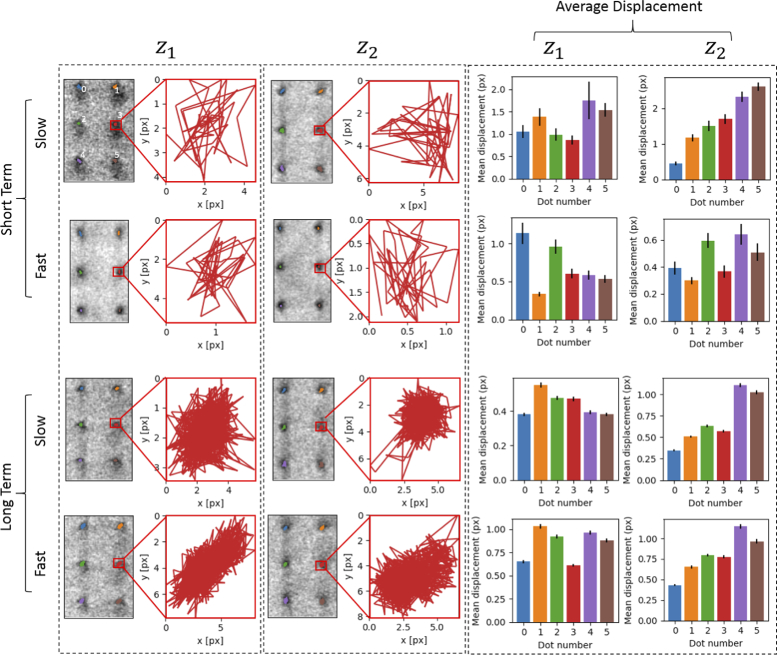
Representative images of a sample of 6 features from the calibration slide imaged at Δz = 15 μm. A custom-written python script tracked and plotted the trajectories of the dots at $z_{1}$ and $z_{2}$ over time. Images were acquired at 10fps or 20fps framerate, for 50 frames (short term) or 5,000 frames (long term). Bar charts represent the mean displacement of each dot (in pixels) calculated from all trajectories, with error bars showing the standard error on the mean.

Figure 8 shows the accuracy and reproducibility of the system to return to the same axial plane position across different sampling schedules and for multiple depth traverses. The displacement maps for 5000 frames per plane appear thicker than those for 50 frames per plane due to the increased number of images acquired. The mean lateral displacement was always ≤ 2 pixels (0.32 μm), which was well within the resolution of the system. There were also no significant or systematic differences in the displacement over different imaging regimes, between axial positions, speed of acquisition or length of acquisition.

### 3.3. Arterial imaging

**Fig. 9.**
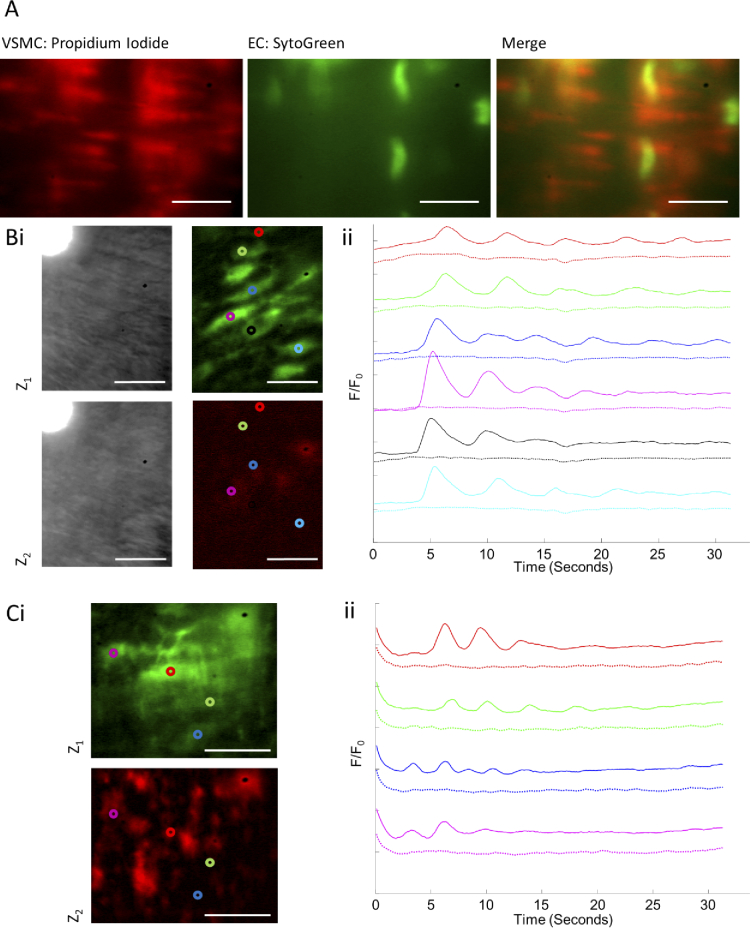
Arterial smooth muscle and endothelial cell layers were imaged sequentially using the epifluorescence remote refocussing system. (A) Fixed carotid arteries stained with PI (1.5 μM) and SytoGreen (5 μM) were imaged on the remote refocussing system at 10fps per plane with an axial separation of 15 μm. VSMCs are shown stained with PI in red, and EC stained with SytoGreen in green. (B) A live carotid artery stained with Cal-520 (5 μM), imaged at 10 fps with an axial separation of 15 μm. (i) The 488 nm and 561 nm laser lines were locked on to the TTL pulse, with the 488 nm laser used to acquire images from ECs ($z_{1}$) and the 561 nm laser to acquire autofluorescence from the unstained VSMC layer ($z_{2}$). Brightfield illumination was turned on at the end of the recording session to verify that both imaging planes were inside the arterial tissue. (ii) Ca^2+^ signals from Cal-520 in ECs were stimulated with ACh (0.5 μM) at 5.5 seconds. Individual Ca^2+^ intensity traces (full lines, colour matched) were plotted as $F/F_{0}$, with corresponding VSMC autofluorescence from the same ROIs (dashed lines). (C) A live carotid artery stained with Cal-520 (5 μM) and TMRE (120 nM), imaged at 10 fps per plane with an axial separation of 15 μm. (i) The 488 nm and 561 nm laser lines were locked on to the TTL pulse, with the 488 nm laser used to acquire images from Cal-520-stained EC Ca^2+^ signals ($z_{1}$) and the 561 nm laser to acquire images from the TMRE-stained VSMC mitochondria ($z_{2}$). (ii) Ca^2+^ signals from Cal-520 in ECs were stimulated with ACh (0.5 μM) at 5.5 seconds. Individual Ca^2+^ intensity traces (full lines, colour matched) were plotted as $F/F_{0}$, with corresponding VSMC mitochondria signal from the same ROIs (dashed lines). Scale bars = 20 μm.

Initial experiments were performed in fixed carotid artery samples to determine whether endothelial cell (EC) and vascular smooth muscle cell (VSMC) layers could be distinguished. Nuclei from both layers were stained with propidium iodide (red) and SytoGreen (green) and imaged using the remote refocus system with the 561 nm and 488 nm laser, respectively. As can be seen in Fig. 9(A), the 561 nm laser accurately imaged the VSMC layer independently of the EC layer, imaged with the 488 nm laser (see Visualization 1). These results show that our system could accurately, rapidly and stably distinguish between these two cell layers.

ECs on live carotid arteries were then stained with the Cal-520 Ca^2+^ indicator dye, and the VSMC layer initially left unstained (Fig. 9(Bi)). Brightfield imaging was also performed at the end of image acquisition to ensure that both $z_{1}$ and $z_{2}$ were within the arterial tissue. ACh is regularly used as an activator and indicator of functional endothelium. Addition of 0.5 μM ACh to the preparation caused characteristic oscillations of Ca^2+^ in endothelial cells imaged with the 488 nm laser in $z_{1}$ (see Visualization 2). These signals were measured at the locations shown by the coloured circles and baseline corrected (*F*/$F_{0}$) to give colour-matched traces of Ca^2+^ activity (Fig. 9(Bii)). To ensure that no signal was being picked up the in 561 nm channel (VSMC autofluorescence), this was subjected to the same analysis at the same locations, and the corresponding traces are shown as dashed lines beneath the 488 nm channel Ca^2+^ traces. The results clearly show that our imaging system can cleanly image the distinct layers without any signal cross over.

To confirm that the signals acquired at $z_{1}$ and $z_{2}$ were distinct and only from the EC and VSMC layers respectively, arterial preparations were stained with Cal-520 Ca^2+^ indicator dye on the EC layer and TMRE mitochondrial indicator on the VSMC layer. EC and VSMC layers were cleanly and distinctly imaged with the 488 nm and 561 nm lasers, respectively (Fig. 9Ci). Analysis of their respective signals after addition of ACh confirmed that no signal from Ca^2+^ oscillations was present in the VSMC layer (Fig. 9Cii).

## 4. Discussion

We have established a system that can image two layers of a sample at high frame rates to quantify activity in endothelial and smooth muscle cells, with a high stability. The high stability of the system may be partly attributed to the steadiness of the moving parts in the refocussing system. Rather than moving large objective lenses, we rely on the movement of a mirror coupled to a motor and a plastic flexure. The flexure limits the movement of the mirror to one dimension, reducing instabilities in other dimensions. The high stability is also maintained at high speed and over long periods of time, with no drift or hysteresis common in other high speed methods of moving mirrors, such as galvanometer mirrors [19]. The speed of the focal switching is, in fact, limited only by the frame rate of the imaging camera and the fluorescent emission levels from the sample. The motor on its own is capable of 4000rpm; however, due to the low motor torque and weight of components in the mechanism, the maximum speed of the system is 183rpm; this could be increased through the use of a motor with a higher torque and lighter components, although these were not necessary for our application.

The maximum camera acquisition rate is a compromise, dependent on a long enough exposure to image the cells with sufficient contrast, but rapid enough to faithfully capture biological activity. It was found that 20 ms was the longest exposure time which could be used at the fastest camera triggering rate, although it is recommended to use lower exposure times. While there is enough light for us to image at a sufficient speed in the current setup, at much higher speeds the beamsplitter in the optical train would need to be replaced with a polarizing beamsplitter and quarter waveplate combination, which would increase the amount of light reaching the camera.

While highly stable, the system is limited somewhat by the difficulty in reducing the distance between the two planes imaged. The axial separation was defined by the user, and the system faithfully recreated the same plane at each pass of the mirror. In this configuration, the minimum axial separation that the optical switches were capable of resolving was 15 μm. Any smaller separation could possibly be achieved using ultra fine adjustment screws (>100 TPI); however, the voltage outputs of the optical switches tend to diminish at very short separations.

The axial resolution of the system when using 60X objectives is 5 μm, which was sufficient to resolve the EC and VSMC layers. Higher numerical aperture lenses could resolve finer separations of the layers, taking into account that refocussing is easier if the second objective lens (${\mathrm{MO}}_{2}$) is an air objective. One must also take into account that the focal plane of the system is always in motion when considering the resolution. For a camera exposure time of 10 ms, for example, the focal plane of the system will move by 3 μm. However, this is lower than the axial resolution of the system and thus does not cause any significant degradation to image quality. The resolution of the system is limited by the numerical aperture of the refocussing objective ${\mathrm{MO}}_{2}$ (0.7), which does not use the full NA of the primary objective ${\mathrm{MO}}_{1}$. This objective was chosen to extend the depth of field of the primary objective, allowing the mirror to travel further which is easier to achieve with the mechanism used. The maximum numerical aperture of the primary objective that we can make use of is approximately 1.05, which is sufficient to resolve the endothelial cell activity. One may argue whether the use of a lower numerical aperture microscope objective as the primary objective would be an advantage for imaging the full depth of the endothelial cells; however, this would greatly reduce the fluorescence detection efficiency, and thus a compromise has been made.

In addition, we have taken advantage of the ability to trigger two excitation lasers separately along with the imaging camera to allow us to completely separate the imaging of cell layers based on the fluorophore used to stain them. This allows us to quantify endothelial cell activity accurately without light from the smooth muscle layer affecting this; meanwhile we may image the smooth muscle layer at the same time to view any changes which take place, without light from the endothelial cell layer interfering.

We have thus developed a refocussing imaging system capable of imaging both the endothelial and smooth muscle cell layers simultaneously with a high stability, using a novel triggering system, providing a convenient and cost effective method for further study of the interplay between the two vascular cell layers.
